# The effects of physiological and injurious hydrostatic pressure on murine *ex vivo* articular and growth plate cartilage explants: an RNAseq study

**DOI:** 10.3389/fendo.2023.1278596

**Published:** 2023-12-07

**Authors:** Lucie E. Bourne, Andrew Hesketh, Aikta Sharma, Giselda Bucca, Peter G. Bush, Katherine A. Staines

**Affiliations:** ^1^ Centre for Lifelong Health, School of Applied Sciences, University of Brighton, Brighton, United Kingdom; ^2^ Department of Mechanical Engineering, University College London, London, United Kingdom

**Keywords:** cartilage, osteoarthritis, hydrostatic pressure, chondrocytes, endochondral ossification, RNAseq

## Abstract

**Introduction:**

Chondrocytes are continuously exposed to loads placed upon them. Physiological loads are pivotal to the maintenance of articular cartilage health, while abnormal loads contribute to pathological joint degradation. Similarly, the growth plate cartilage is subject to various loads during growth and development. Due to the high-water content of cartilage, hydrostatic pressure is considered one of the main biomechanical influencers on chondrocytes and has been shown to play an important role in the mechano-regulation of cartilage.

**Methods:**

Herein, we conducted RNAseq analysis of ex vivo hip cap (articular), and metatarsal (growth plate) cartilage cultures subjected to physiological (5 MPa) and injurious (50 MPa) hydrostatic pressure, using the Illumina platform (n = 4 replicates).

**Results:**

Several hundreds of genes were shown to be differentially modulated by hydrostatic pressure, with the majority of these changes evidenced in hip cap cartilage cultures (375 significantly upregulated and 322 downregulated in 5 MPa versus control; 1022 upregulated and 724 downregulated in 50 MPa versus control). Conversely, fewer genes were differentially affected by hydrostatic pressure in the metatarsal cultures (5 significantly upregulated and 23 downregulated in 5 MPa versus control; 7 significantly upregulated and 19 downregulated in 50 MPa versus control). Using Gene Ontology annotations for Biological Processes, in the hip cap data we identified a number of pathways that were modulated by both physiological and injurious hydrostatic pressure. Pathways upregulated in response to 50 MPa versus control, included those involved in the generation of precursor metabolites and cellular respiration. Biological processes that were downregulated in this tissue included ossification, connective tissue development, and chondrocyte differentiation.

**Discussion:**

Collectively our data highlights the divergent chondrocyte phenotypes in articular and growth plate cartilage. Further, we show that the magnitude of hydrostatic pressure application has distinct effects on gene expression and biological processes in hip cap cartilage explants. Finally, we identified differential expression of a number of genes that have previously been identified as osteoarthritis risk genes, including Ctsk, and Chadl. Together these data may provide potential genetic targets for future investigations in osteoarthritis research and novel therapeutics.

## 1 Introduction

Articular cartilage is a specialized connective tissue that covers the ends of bones in synovial joints and facilitates joint movement. It is load bearing and therefore protects underlying subchondral bone from excessive forces. The articular cartilage consists of chondrocytes which retain a stable phenotype to ensure the longevity of the tissue (1, 2). This is in contrast to the chondrocytes of the growth plate cartilage which undergo defined stages of maturation and differentiation to enable longitudinal bone growth (3).

Structurally, the articular cartilage can be divided into superficial, intermediate, and deep zones which are distinct in their organization of both the chondrocytes, surrounded by their individual pericellular matrix, and the collagen type-II and aggrecan-rich matrix (3). The articular cartilage functions to withstand physiological loading over the life-course. However, in the degenerative joint disease osteoarthritis, pathology is characterized by progressive articular cartilage degradation (4). Whilst osteoarthritis is well established to affect all tissues of the joint, the cellular and molecular mechanisms are incompletely understood (5–7). Various forms of mechanical stimuli are involved in the maintenance of the articular cartilage and thus the mechanoresponse of the chondrocyte plays an important role in the development of osteoarthritis (8–10). Compression, tensile and shear stress result in deformative loading, whereas osmotic and hydrostatic pressure induce stress without tissue or cellular deformation (8, 9, 11, 12). As a highly hydrated tissue, interstitial fluid pressurization within the articular cartilage is considered one of the main biomechanical influencers on chondrocytes (13–15). Throughout the cartilage zones, chondrocytes are subjected and respond to a hydrostatic pressure gradient, ranging from 0.1-10 MPa, to direct matrix remodeling, chondrogenesis and chondrocyte metabolism (13, 14). However, excessive hydrostatic pressure (≥20 MPa) outside the physiological range has been shown to induce apoptosis, alter cell morphology and metabolism, reduce extracellular matrix (ECM) synthesis, induce inflammatory cytokine production, and modulate oxidative stress (16–19).

*In vitro*, hydrostatic pressure can be applied experimentally to cells and tissues derived from both animals and humans to investigate mechanotransduction, for example in monolayer cultures (20–23), micromass or pellet cultures (24, 25), 3D cell scaffolds (26–29), and explant cultures (17, 22, 30, 31). The ability to provide either dynamic or continuous hydrostatic pressure, alter the magnitude and/or the duration of pressure provides an alternative approach to study the effects of mechanical stimulation (13, 32). Whilst there is little consensus within the field on the duration and pressure magnitudes in cultures, our previous meta-analysis has indicated that in human and animal- derived cells, low pressure (5 MPa) leads to anabolic responses, including elevated aggrecan expression and proteoglycan release, whereas a higher pressure (50 MPa) has a negative effect on proteoglycan production (33). Therefore, it is possible to investigate the effects of hydrostatic pressure at both physiological and pathophysiological levels.

To determine the effects of hydrostatic pressure on the molecular pathways involved in the regulation of chondrocyte physiology, transcriptomic analyses are often employed to identify responsive genes. Several studies in animal cells have utilized these approaches in the study of chondrocyte progenitor cells, immortalized chondrocytes, and primary chondrocytes within a hydrogel; however, transcriptome sequencing on *ex vivo* models has not yet been performed (21, 29, 34). Phenotypic changes are often observed in cells cultured in a monolayer, with cells de-differentiating or altering morphology, whereas *ex vivo* models allow examination of cells within their native environment (35). Herein, the aim of this study was to perform RNAseq analysis on two murine *ex vivo* cartilage models (hip cap and metatarsal) after exposure to physiological and injurious hydrostatic pressure, to examine the effects of hydrostatic pressure on gene expression in two different chondrocyte phenotypes.

## 2 Methods

### 2.1 Isolation and culture of ex vivo cartilage models

All mice utilized in these studies were kept in controlled conditions at the University of Brighton and all tissue isolation procedures were performed in accordance with the UK Animals (Scientific Procedures) Act of 1986 and regulations set by the UK Home Office and local institutional guidelines (PPL: PP3310437). Analyses were conducted blindly where possible to minimize the effects of subjective bias. Animal studies were conducted in line with the ARRIVE guidelines.

**Figure 1:**
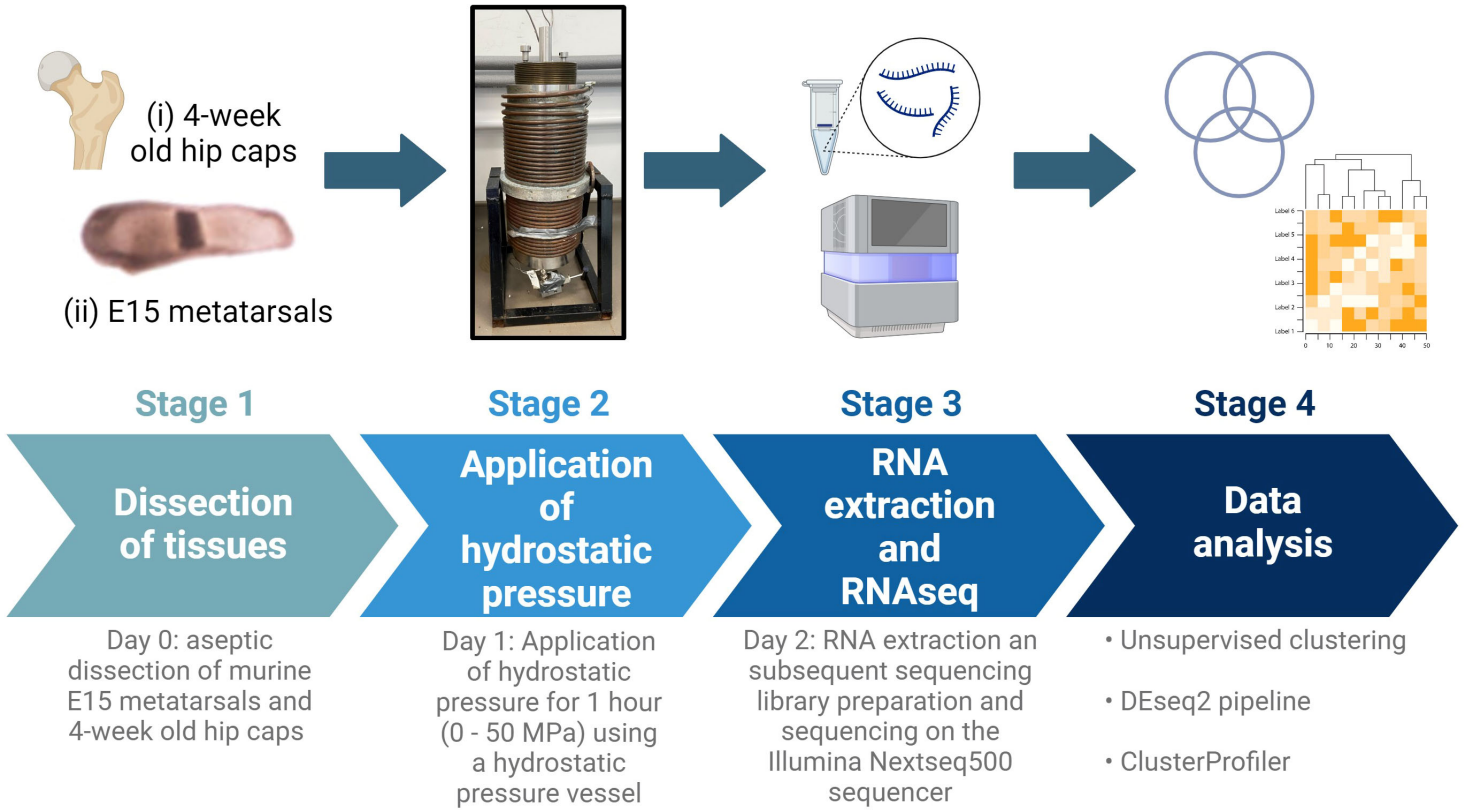
Schematic of experimental design. E15 metatarsal bones and 4-week-old hip cap cartilage explants were subjected to hydrostatic pressure (0-50 MPa) for 1 hour. After 24 hours, RNA was extracted and RNAseq and downstream analyses conducted. Created with BioRender.com.

Femoral heads were isolated from 4-week-old male C57/BL6J mice (Charles River), as previously described (**Figure 1**) (36). In brief, the hip joint was dislocated by applying slight pressure at the joint, and the femoral cap was avulsed using forceps. At this developmental stage, the predominant component of this tissue is the articular cartilage, therefore underlying subchondral bone was not included. Both hip caps were pooled from each individual mouse (n=4 mice/experimental group). Hip caps were cultured in Dulbecco’s Modified Eagle Medium with GlutaMAX, substituted with 100 U/ml penicillin, 100µg/ml streptomycin (Thermo Fisher Scientific) in a humidified atmosphere (37°C, 5% CO_2_).

Embryonic metatarsal organ cultures provide a well‐established model of endochondral bone growth (**Figure 1**) (37). Metatarsals were isolated from E15 embryos of C57/BL6J (Charles River) mice as previously described (36). Six metatarsal bones were pooled per sample (n=4 samples/experimental group). Metatarsal bones were cultured in α-Minimum Essential Medium supplemented with 0.2% BSA Fraction V; 1 mmol/l β-glycerophosphate (βGP); 0.05 mg/ml L-ascorbic acid phosphate; 0.05 mg/ml gentamicin and 1.25 μg/ml fungizone (Thermo Fisher Scientific) in a humidified atmosphere (37°C, 5% CO_2_).

### 2.2 Application of hydrostatic pressure

After 24 hours of culture, hips caps and metatarsals were placed into 5 ml sterile plastic syringes fitted with Luer lock end caps, taking care to eliminate all air bubbles (**Suppl. Figure 1**). Movement of the syringe plunger allowed for equilibration of pressure between syringe contents and the pressure vessel water (17). The syringes were placed in a water-filled pressure vessel at room temperature. Syringes were pressurized to 0 MPa (control), 5 MPa (physiological) or 50 MPa (injurious) hydrostatic pressure for 1 hour (**Figure 1**; **Suppl. Figure 1**). Following exposure to hydrostatic pressure, tissues were placed back into the incubator and cultured for a further 24 hours in the respective media, then flash frozen at -80°C until RNA extraction.

### 2.3 RNA extraction and sequencing

Tissue (<100 mg) were defrosted on ice and 1 ml Trizol (Qiagen) was added to each sample; tissues were homogenised using a mechanical disruptor, making sure to keep them cool by putting on ice every 15 seconds. Samples were incubated at room temperature for a minimum of 10 minutes to allow for cell lysis and centrifuged at 12,000 x *g* for 15 minutes at 4°C to pellet the excess tissue, whilst retaining RNA in solution. The supernatant was transferred to a clean tube and 200 μL of chloroform (Sigma) added. After vigorous shaking for 20 seconds, the samples were incubated at room temperature for 3 minutes and then centrifuged at 12,000 x *g* for 15 minutes at 4°C to enable phase separation. The upper, aqueous phase was transferred to a new tube, avoiding the interface. Following the addition of an equal volume of 70% ethanol, the samples were mixed thoroughly by vortexing and total RNA purified using RNeasy Mini spin columns (Qiagen), according to the manufacturer’s recommendations. Purified RNA was eluted in 30 μl of RNase-free water, repeating the elution twice by reapplying the elute. The concentration and purity of the RNA samples were assessed using a Nanodrop One C spectrophotometer (Labtech) and the quality of the RNA was assessed on a TapeStation 4200 (Agilent Technologies).

All samples passed purity quality control checks but exhibited RNA Integrity Number (RIN) equivalent values below the ideal minimum of 7 (average value 2.8). The low RIN values obtained are considered typical for these explant tissue samples and suggest some partial degradation of the total RNA. DV200 analysis using the Agilent TapeStation 4200 software showed a percentage of fragments between 200 and 10000 bp ranging between 53.51-87.4% in all RNA samples. Sequencing libraries were prepared using the Universal Plus™ Total RNASeq with NuQuant kit and a mouse rRNA depletion module (Tecan Genomics), required for partially degraded RNA samples. Library construction strategy was pair end and strand specific. Libraries were checked for quality using the TapeStation 4200, quantified, normalized and sequenced on the Illumina NextSeq500 sequencer using a high-output kit (17 libraries) and a mid-output kit (7 libraries).

### 2.4 Data analysis

Initial sequencing read quality control was conducted using fastqc (version 0.11.9) (38) and multiqc (version 1.8) (39). Trimming was performed using TrimGalore using a minimum quality threshold of 20, discarding any trimmed reads shorter than 20 nucleotides. Trimmed reads were quantified using kallisto quant and transcript quantifications were converted to gene level by tximport. The transcriptome mapping data for all samples was imported into R for data summarization at the gene level. The data was normalized and analyzed using the DESeq2 pipeline (40). Unsupervised clustering of the sample data was performed using the R packages pheatmap and pcaMethods. Significant genes were identified by analysis using a model design that considered the sequencing run and strandedness of the library as possible batch effects (design= ~SeqRun + Library + Condition) and applying a 5% significance threshold to p-values adjusted using the Benjamini and Hochberg procedure (a significance threshold referred to elsewhere in the text as padj<=0.05, or 5% FDR). For functional analysis of the groups of differentially expressed genes, clusterProfiler was utilized to identify significantly over-represented functional categories using a significance threshold of 5% on the Benjamini and Hochberg corrected p-values (41). Annotations for the Gene Ontology (GO) Biological Process (BP), from the R package org.Mm.eg.db (version 3.11.4) were used (42). Genes that were significantly differentially expressed between our samples were compared to recent genome-wide association studies of osteoarthritis that have identified a number of osteoarthritis risk genes (43, 44).

## 3 Results

**Figure 2:**
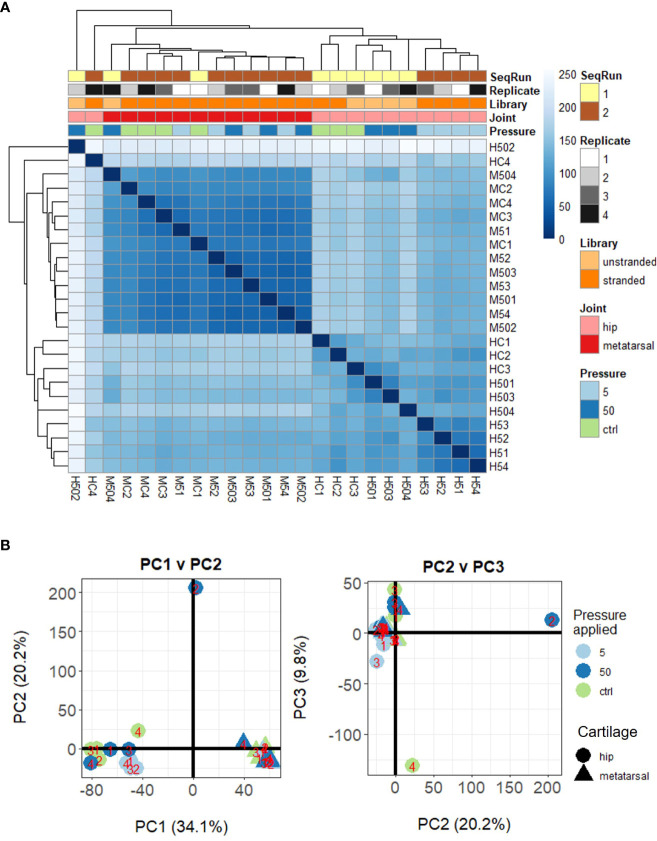
Unsupervised clustering of samples based on their DESeq2 normalized gene-level counts. **(A)** Heat map of inter-sample Euclidean distances, where darker blue colors indicate closer similarity. **(B)** Principal components analysis. Samples are labelled as M (metatarsal) and H (hip cap), followed by C (control – 0 MPa), 5 (5 MPa) or 50 (50 MPa) and replicate number (1–4).

Herein, we conducted RNAseq analysis of murine *ex vivo* hip cap (articular), and metatarsal (growth plate) cartilage cultures (n=4 replicates) subjected to physiological (5 MPa) and injurious (50 MPa) hydrostatic pressure. Unsupervised clustering of the gene expression data indicated a clear distinction between the hip cap and metatarsal sample data, but two of the hip cap cartilage samples (*H502* [exposed to 50 MPa hydrostatic pressure] and *HC4* [control, 0 MPa hydrostatic pressure]) appeared to be outliers, thus were excluded from all downstream statistical analyses (**Figures 2A, B**; **Suppl. Figures 2, 3**).

### 3.1 Gene expression profiles of articular and growth plate cartilage

**Figure 3:**
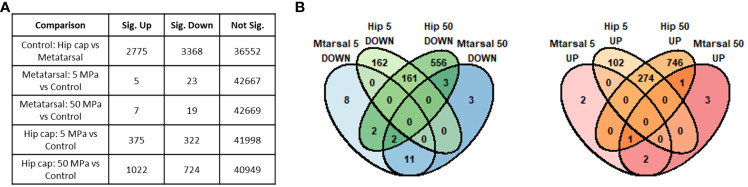
Summary of genes identified as significantly differently expressed between the main sample conditions of interest (DESeq2 padj<=0.05 (5% FDR)). **(A)** Numbers of significant genes (padj<=0.05) in each comparison. Sig. Down indicates genes down-regulated in the first condition listed in the comparison column relative to the second, while Sig. Up indicates those up-regulated in the first condition. **(B)** Overlap between the significant genes identified in each hip cap (Hip) and metatarsal (Mtarsal) cartilage explant group.

Prior to differential gene expression analyses focusing on the effects of hydrostatic pressure, the gene expression profiles of the two different cartilage explants were investigated to assess the genes and pathways that may be differentially expressed between a transient (growth plate) and an inherently stable (articular) cartilage phenotype (**Suppl. Data 1**). There were 2775 genes upregulated and 3368 genes downregulated in hip cap cartilage in comparison to metatarsal cartilage (**Figure 3A**). Upregulated genes with the greatest log_2_ fold change included ribosomal protein L9 (*Rpl9-ps4*, 39.4-fold), collagen type X (*Col10a1*, 7.7-fold), and frizzled-related protein (*Frzb*, 7.2-fold) (**Table 1**). Downregulated genes with the greatest log_2_ fold change included microfibrillar-associated protein 4 (*Mfap4*, 8.6-fold), insulin-like growth factor binding protein 2 (*Igfbp2*, 7.3-fold) and fibroblastic growth factor 10 (*Fgf10*, 7.2-fold) (**Table 1**).

**Table 1:** Top 10 genes with highest upregulation and top 10 genes with highest downregulation in the hip cap versus metatarsal RNAseq datasets.

	Gene Name	Log_2_ Fold Change	Adjusted *p* value
Top 10 genes with highest upregulation	*Rpl9-ps4*	39.41761815	6.53953E-15
	*Gm10925*	23.98054338	1.87468E-05
	*Gm22969*	21.45491466	5.33958E-06
	*Mif-ps4*	14.34623609	1.37344E-05
	*Col10a1*	7.736184458	3.71322E-08
	*Serpina1d*	7.646536307	5.54893E-13
	*Frzb*	7.219881296	2.58347E-50
	*Cytl1*	7.218161967	2.07064E-45
	*Gpx3*	7.055584365	1.53747E-28
	*Clec3a*	7.031246207	4.9778E-14
Top 10 genes with highest downregulation	*Mfap4*	-8.564081588	4.07328E-34
	*Xist*	-8.407700367	5.36182E-12
	*Actc1*	-8.087469882	1.48585E-10
	*Hoxd13*	-7.737458742	1.44143E-30
	*Myh3*	-7.539165841	9.66177E-24
	*Kera*	-7.465016823	2.52732E-12
	*Igfbp2*	-7.34202703	1.197E-12
	*Crabp1*	-7.311799886	8.05824E-13
	*Fgf10*	-7.176158119	4.15686E-11
	*Ptn*	-7.059869481	1.53489E-97

Next, we sought to examine whether these differentially expressed genes were enriched in particular biological processes. Using annotations for GO BP, the data revealed a number of significantly enriched processes, which include ossification (GO:0001503; 124 genes), bone development (GO:0060348; 81 genes), cartilage development (GO:0051216; 83 genes), connective tissue development (GO:0061448; 98 genes), and extracellular matrix organization (GO:0030198; 84 genes), in hip cap cultures in comparison to metatarsals (**Suppl. Table 1**). Conversely, those that were downregulated included muscle tissue development (GO:0060537; 140 genes) and muscle cell differentiation (GO:0042692; 127 genes), as well as synapse organization (GO:0050808; 142 genes) (**Suppl. Table 1**).

When comparing the two datasets, the hip cap data yielded many more significant changes than the metatarsal data, and the greater spread of log_2_ fold changes taking place in the hip cap samples suggests that the hip cap cartilage explants are more responsive to changes in pressure than the metatarsal explants (**Figure 3**). Therefore, subsequent analyses focused on the data from the hip cap explants, with the highest up- and down-regulated genes, either commonly or uniquely expressed between each group, in the metatarsal data sets detailed in **Suppl. Tables 2, 3**.

### 3.2 Effects of physiological and injurious hydrostatic pressure on gene expression in hip cap cartilage explants

Compared to control, there were 375 genes significantly upregulated with 5 MPa hydrostatic pressure and 322 significantly downregulated in hip cap cultures (**Figure 3A**; **Suppl. Data 1**). With injurious hydrostatic pressure (50 MPa), there were 1022 significantly upregulated and 724 significantly downregulated genes (**Figure 3A**; **Suppl. Data 1**). Whilst some of the genes were consistently up- or down-regulated across the two hydrostatic pressures in both the hip cap and the metatarsal datasets, most of these significant genes were uniquely expressed by the hip cap datasets at 5 or 50 MPa (**Figure 3B**). Genes commonly modulated by hydrostatic pressure in these cultures with the greatest log_2_ fold changes are detailed in **Suppl. Table 4**. The genes uniquely expressed in response to hydrostatic pressure magnitudes include *Car2* (upregulated in 5 MPa versus control, 1.5-fold), *Mlip* (upregulated in 50 MPa vs control, 2.6-fold), *Tg* (downregulated in 5 MPa versus control, 2.2-fold) and *Ryr3* (downregulated in 50 MPa versus control, 2.6-fold) (**Table 2**).

**Table 2:** Top 10 genes with highest upregulation and greatest downregulation that are uniquely expressed in 5 MPa versus control and 50 MPa versus control in the hip cap RNAseq datasets.

	5 MPa vs Control			50 MPa vs Control		
	Gene Name	Log_2_ Fold Change	Adjusted *p* value	Gene Name	Log_2_ Fold Change	Adjusted *p* value
Top 10 genes with highest upregulation	*Abhd15*	3.22895414	0.000519	*Gm2451*	18.04382867	1.98582E-05
	*Car2*	1.466531813	0.008049	*H2ac23*	15.91830736	0.000179818
	*Gm45665*	1.436157039	0.00787	*Gm9973*	3.311225437	5.25837E-08
	*Olfr1380*	1.18544216	0.033261	*Gm48942*	3.201576037	3.37509E-06
	*Mpp5*	1.14918265	0.000377	*Gm29408*	3.061996469	4.83774E-06
	*Gm9962*	1.142757676	0.013066	*Mlip*	2.567534817	0.000385784
	*Fpr1*	1.061193105	0.016204	*Mmp12*	2.501682447	3.10048E-05
	*Atp5g2*	1.039789631	0.00418	*Abcd2*	2.312011543	0.001575342
	*Fam81a*	1.004756565	0.011008	*Ywhaq-ps3*	2.243694849	0.046074893
	*Alg8*	0.977342633	0.001856	*mt-Nd6*	2.195320542	0.001507966
Top 10 genes with highest downregulation	*Rps18-ps6*	-3.33818292	0.005493	*Gm44732*	-4.24186258	0.000124443
	*Gm23680*	-3.158700551	0.000322	*Gm16479*	-3.327634912	0.000538299
	*Gm9968*	-3.054024647	0.000146	*Ryr3*	-2.596299426	0.002870783
	*Gm24514*	-2.45810073	1.17E-06	*Gm3625*	-2.176518378	1.83627E-05
	*Tg*	-2.18679795	0.001011	*Pla2g2c*	-2.166700674	0.046074893
	*Gm25682*	-1.92383803	0.003421	*Gm8249*	-1.940743018	0.002267981
	*Gm42715*	-1.658526363	0.000376	*Serpina1a*	-1.854010245	0.014092563
	*Adgrb1*	-1.380005988	0.005428	*H2-M5*	-1.718905493	0.003710939
	*Gm26822*	-1.305512292	0.001159	*Gm15807*	-1.69503207	0.003989171
	*Rap1gap2*	-1.246295417	0.012053	*Serpina1d*	-1.649670074	0.000777013

### 3.3 GO BP enrichment analysis of differentially expressed genes

**Figure 4:**
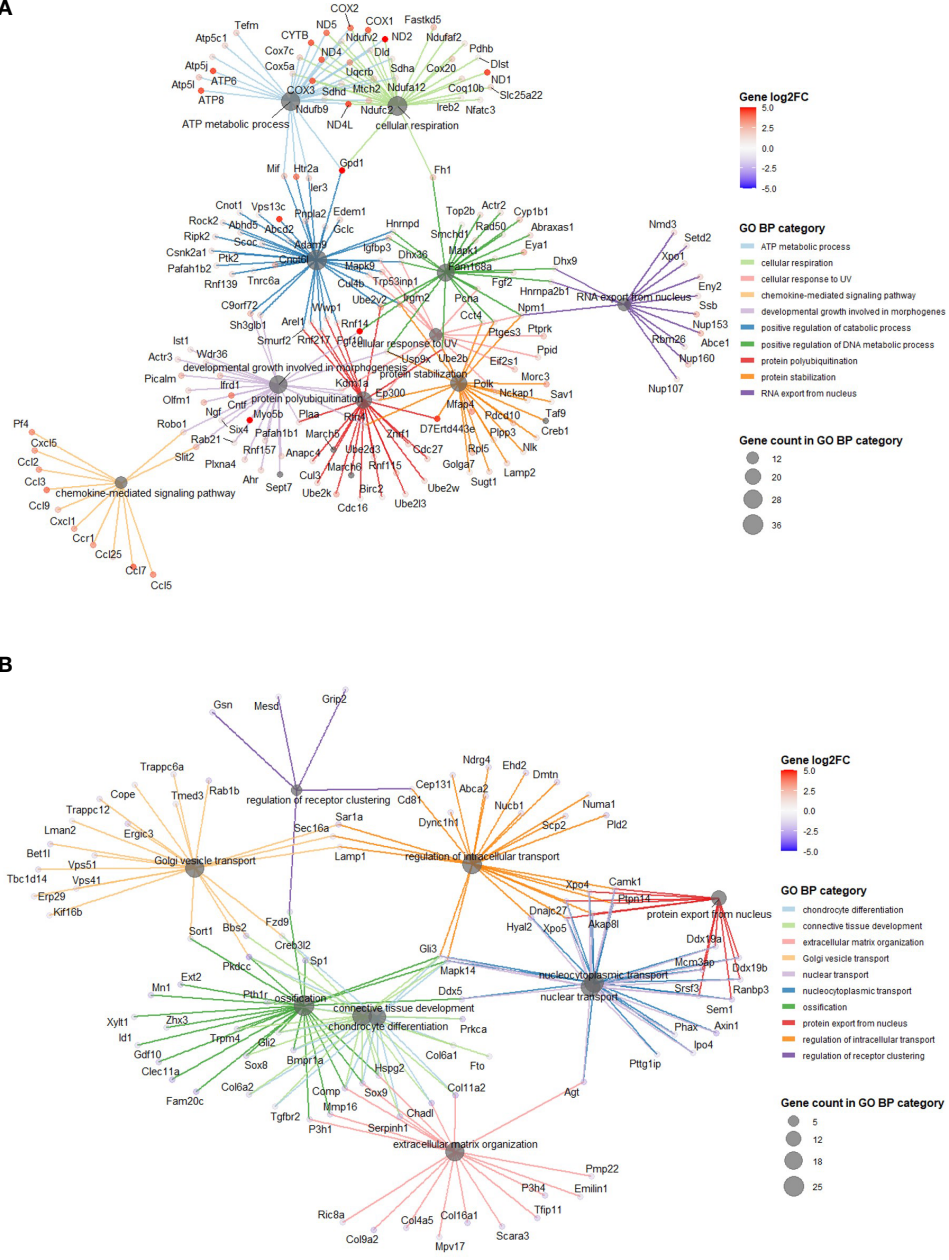
GO BP categories in 50 MPa versus control hip cap datasets. **(A)** Pathways enriched in the genes significantly up in the 50 MPa versus control comparisons. **(B)** Pathways enriched in the genes significantly down in the 50 MPa versus control comparisons.

Using annotations for GO BP, the data revealed significantly enriched processes including regulation of cytokine production (GO:001819; 21 genes), Ras protein signal transduction (GO:007265; 20 genes) and ATP metabolic processes (GO0046034; 18 genes) with 5 MPa hydrostatic pressure application (**Table 3**; **Suppl. Data 2**). Conversely, process including cellular component disassembly (GO:0022411; 16 genes) and nuclear transport (GO0051169; 15 genes) were downregulated (**Table 3**; **Suppl. Data 2**). With injurious hydrostatic pressure (50 MPa), enriched pathways included generation of precursor metabolites and energy (GO:0006091; 39 genes), and cellular respiration (GO:0045333, 31 genes) (**Figure 4**; **Table 3**; **Suppl. Data 2**). Other upregulated GO BP relevant to the known functions of chondrocytes included regulation of developmental growth (GO0048638; 33 genes), and regulation of cell size (GO0008361; 21 genes) (**Suppl. Data 2**). Whereas those downregulated included ossification (GO:0001503; 25 genes), cartilage development (GO:0051216; 18 genes), connective tissue development (GO:0061448; 21 genes), and chondrocyte differentiation (GO:0002062; 17 genes) (**Table 3**; **Suppl. Data 2**). Further analysis of these enriched pathways in injurious hydrostatic pressure highlighted differential expression of several genes known to be involved in osteoarthritis, such as *Fgf2, Ep300, Ngf, Adam9, Igfbp3, Sox9, Comp, Col6a1, Col6a2* and *Col11a1*.

**Table 3:** Annotations for the Gene Ontology (GO) Biological Process (BP) for genes that are differentially expressed in 5 MPa versus control and 50 MPa versus control in the hip cap RNAseq datasets.

5 MPa vs Control				
	ID	Description	No. genes	Adjusted *p* value
Top 10 upregulated GOBP	GO:0001819	positive regulation of cytokine production	21	1.38E-06
	GO:0007265	Ras protein signal transduction	20	1.50E-06
	GO:0046034	ATP metabolic process	18	1.05E-08
	GO:0045333	cellular respiration	17	1.39E-10
	GO:0015980	energy derivation by oxidation of organic compounds	17	1.11E-07
	GO:0022904	respiratory electron transport chain	13	8.59E-11
	GO:0022900	electron transport chain	13	1.61E-10
	GO:0042773	ATP synthesis coupled electron transport	12	3.79E-11
	GO:0006119	oxidative phosphorylation	12	9.95E-09
	GO:0042775	mitochondrial ATP synthesis coupled electron transport	9	8.31E-08
Top 10 downregulated GOBP	GO:0022411	cellular component disassembly	16	3.92E-06
	GO:0006913	nucleocytoplasmic transport	15	1.31E-06
	GO:0051169	nuclear transport	15	1.31E-06
	GO:0033157	regulation of intracellular protein transport	11	3.94E-05
	GO:0051168	nuclear export	10	3.58E-06
	GO:0015931	nucleobase-containing compound transport	10	4.64E-05
	GO:0006611	protein export from nucleus	9	1.07E-05
	GO:0034453	microtubule anchoring	5	1.01E-05
	GO:0018023	peptidyl-lysine trimethylation	5	0.000157
	GO:0034454	microtubule anchoring at centrosome	3	0.000166
50 MPa vs Control				
	ID	Description	No. genes	Adjusted *p* value
Top 10 upregulated GOBP	GO:0006091	generation of precursor metabolites and energy	39	5.16E-09
	GO:0015980	energy derivation by oxidation of organic compounds	34	2.82E-11
	GO:0045333	cellular respiration	31	1.64E-14
	GO:0046034	ATP metabolic process	29	1.28E-08
	GO:0022904	respiratory electron transport chain	22	4.28E-14
	GO:0022900	electron transport chain	22	1.27E-13
	GO:0006119	oxidative phosphorylation	20	1.47E-10
	GO:0042773	ATP synthesis coupled electron transport	19	1.49E-13
	GO:0042775	mitochondrial ATP synthesis coupled electron transport	16	7.34E-11
	GO:0009060	aerobic respiration	14	2.61E-07
Top 10 downregulated GOBP	GO:0001503	ossification	25	5.11E-05
	GO:0032386	regulation of intracellular transport	23	6.10E-05
	GO:0061448	connective tissue development	21	3.88E-05
	GO:0006913	nucleocytoplasmic transport	21	4.75E-05
	GO:0051169	nuclear transport	21	4.75E-05
	GO:0048193	Golgi vesicle transport	20	3.78E-05
	GO:0051216	cartilage development	18	2.02E-05
	GO:0002062	chondrocyte differentiation	17	2.76E-08
	GO:0051168	nuclear export	13	8.25E-05
	GO:1903909	regulation of receptor clustering	5	9.33E-05

### 3.4 Differential expression of previously identified osteoarthritis risk genes

Recent genome-wide association studies of osteoarthritis have identified a number of osteoarthritis risk genes (43, 44). We therefore sought to compare whether these genes were differentially expressed in response to hydrostatic pressure in our datasets (**Table 4**). Only one of these genes was differentially expressed in our 5 MPa versus control datasets (*Wscd2*, 0.6-fold downregulation; data not shown). However, with injurious (50 MPa) hydrostatic pressure application, there were 12 genes differentially expressed (**Table 4**). These included cathepsin K (*Ctsk*, 0.9-fold upregulation), and chondroadherin-like (*Chadl*, 0.9-fold downregulation) (**Table 4**; **Suppl. Figure 4**).

**Table 4:** Differential expression of osteoarthritis risk genes identified in recent genome-wide association studies in response to injurious hydrostatic pressure (50 MPa) versus control in our hip cap datasets.

	Gene Name	Log_2_ Fold Change	Adjusted *p* value
Upregulated genes	*Ctsk*	0.852667719	0.016121811
	*Il11*	0.750247415	0.036698304
	*Sbno1*	0.740095825	0.005159578
	*Aldh1a2*	0.648544373	0.037375403
Downregulated genes	*Chadl*	-0.904205798	0.009053645
	*Apoe*	-0.899057162	0.001558965
	*Mn1*	-0.790048116	0.020283481
	*Pfkm*	-0.736351338	0.017447093
	*Megf8*	-0.647300732	0.001249084
	*Fto*	-0.492563212	0.035580843
	*Vgll4*	-0.490894811	0.049748082
	*Smg6*	-0.47338466	0.043509481

## 4 Discussion

In this study we conducted RNAseq analysis of two different *ex vivo* cartilage explants (metatarsal and hip cap), to examine the effects of two magnitudes of hydrostatic pressure on gene expression. We observed clear differences between the cartilage types, including the upregulation of key genes such as *Frzb* and *Col10a1* in the hip cap explants. Extensive changes in gene expression were observed with hydrostatic pressure in the hip cap cartilage groups, however this was to a weaker extent in the metatarsal explants. Within the hip cap data set, enriched GO BP in the genes that were significantly downregulated in response to injurious hydrostatic pressure (50 MPa) versus control, included those involved in cartilage, bone and connective tissue development. Interestingly, these pathways were also increased when comparing the hip cap to the metatarsal data, suggesting that injurious hydrostatic pressure may promote a more transient-like phenotype in the hip cap cultures. This is further supported by our observed enrichment of the GO BPs for developmental growth and cell size in hip caps exposed to 50 MPa hydrostatic pressure. Indeed, it is well established that in osteoarthritis, the inherently stable articular cartilage undergoes changes that reflect a more developmental cartilage phenotype, such as that in the growth plate (3, 7). Therefore, lessons can be learnt from a better understanding of these two phenotypes, and their similarities and differences in our pursuit of maintaining articular cartilage health in ageing. This is of particular importance given the lack of regenerative capability of the articular cartilage, thus meaning therapies for osteoarthritis remain limited (4, 7).

Articular cartilage covers the ends of the bones in synovial joints, and the chondrocytes within maintain a stable phenotype to ensure joint health and longevity. This is in contrast to the growth plate cartilage, which is more transient in nature, with chondrocytes undergoing differentiation processes which drive endochondral ossification and longitudinal bone growth (3). The chondrocytes of these two cartilaginous structures express different programs, further defined by our RNAseq analysis in hip cap (articular) and metatarsal (growth plate) cartilage. Amongst the most differentially expressed genes in our studies were *Frzb*, and *Col10a1* (both upregulated) and *Igfbp2*, and *Fgf10* (both downregulated). *Col10a1* is a key determinant of chondrocyte hypertrophy, with mutant or abnormal human *Col10a1* expression associated with abnormalities in this process (45–47). The increase in *Col10a1* in our hip cap explants therefore suggests a greater degree of hypertrophy than in our metatarsal explants. Abnormal *Col10a1* expression is a well-established feature in osteoarthritis (48–50). Similarly, two SNPs in *Frzb*, an antagonist of the canonical WNT pathway, have been associated with osteoarthritis (51–53). Further, in pre-clinical models, osteoarthritis severity scores are significantly higher in the joints with deletion of *Frzb* compared to littermates (54). Together, our data are consistent with previous studies considering the different phenotypes of these cells, thus suggesting diverging phenotypes of these cell populations (55–57).

The high-water content of cartilage (approx. 70-80% water per wet mass) is maintained by an abundance of proteoglycans in the matrix. Chondrocytes in both the growth plate and the articular cartilage are subjected to a number of mechanical forces, including compressive and shear stresses, during loading (9, 13). These mechanical signals then modulate biochemical activity and changes in chondrocyte behavior (22). The majority of research to date has focused on understanding compressive forces on the health of the articular cartilage, however most of this force transforms to hydrostatic pressure due to the interstitial fluid content of joints (14, 58). As such, it can be assumed that hydrostatic pressure is the more prevalent stress to which chondrocytes are exposed. Chondrocytes demonstrate an improved cartilaginous physiology when exposed to hydrostatic pressure, as indicated by their increased ECM production (13). This therefore suggests that understanding the complexities of hydrostatic pressure could be a potential avenue for tissue regeneration in osteoarthritis.

Despite the application of hydrostatic pressure being experimentally controllable, studies have varied in their magnitude, style and duration of hydrostatic pressure application. Our previous meta-analysis informed these factors in the experimental set up for our RNAseq study herein (33). In articular cartilage during normal movement, typical hydrostatic pressure loading of 0.5–10 MPa have been measured (13, 59). Our meta-analysis in 3D cultured chondrocytes confirmed that, based on aggrecan gene expression data, 4–5 MPa can significantly enhance proteoglycan production (33). Conversely, our meta-analysis detailed that the hydrostatic pressure magnitude of 50 MPa had a negative effect on proteoglycans (33). As such, we deemed the magnitudes of physiological (5 MPa) and injurious (50 MPa) hydrostatic pressure to be applicable in our pursuit of understanding gene changes in our explants.

In an RNAseq study performed on monolayer cultures, Zhu et al. used human articular chondrocytes to compare hydrostatic pressure (0.1 MPa) and perfusion methods on the chondrocyte phenotype, with the aim of understanding methods for reducing chondrocyte dedifferentiation in culture (60). Their RNAseq analysis revealed upregulation of well-known chondrocyte genes with hydrostatic pressure and conclude that a low hydrostatic pressure can be beneficial to chondrocytes (60). Further, a previous microarray study examined the effects of continuous hydrostatic pressure (25 MPa) on the chondrogenic ATDC5 cell line, again cultured in monolayer (21). Similarities can be observed between the genes they observe to be modulated by hydrostatic pressure and ours described herein, including differential expression of apoptosis-related and cartilage matrix genes (21). However, Montagne et al. applied a continuous hydrostatic pressure for 24 hours, which is in comparison to our study whereby we applied a single load for 1 hour and is akin to a single injurious event. Further, our examination of two different magnitudes of hydrostatic pressure and in physiologically-relevant cartilage explants adds further strength to our study. In addition, several genes known to play a key role in progression of osteoarthritis (e.g., *Fgf2, Ep300, Ngf, Adam9, Igfbp3, Sox9, Comp, Col6a1, Col6a2* and *Col11a1*) were modulated in our injurious hydrostatic pressure hip cap datasets, thereby validating this approach.

Overall, our results seem to indicate osteoarthritic-like effects of injurious hydrostatic pressure on our hip cap cartilage explants. Among the modulated genes identified in our study, several genes which have been identified as osteoarthritis risk genes from recent GWAS studies were differentially expressed, however verification of these by *in situ* hybridization or RT-qPCR would be beneficial (43, 44). There was only one gene (*Wscd2*, WSC Domain-Containing Protein 2) modulated in the 5 MPa versus control dataset, with the majority being in the 50 MPa comparison. Interestingly, *Wscd2* has previously been identified as an osteocyte transcriptome signature gene and downregulated in murine bone with ageing, although its role in cartilage has, to our knowledge, not yet fully been defined (61, 62).

Of these risk genes modulated by 50 MPa hydrostatic pressure, the gene that underwent the highest fold upregulation was cathepsin K (*Ctsk*), a protein expressed by osteoclasts used for collagen degradation (63). This finding is consistent with the previous microarray study by Montague et al. in which *Ctsk* was found to be strongly induced following the exposure of hydrostatic pressure for 4 hours (21). Indeed, *Ctsk* has been shown to be overexpressed in the articular cartilage and subchondral bone in osteoarthritis (64, 65). Further, *Ctsk* deletion in a murine surgical osteoarthritis model (destabilization of the medial meniscus) protected against disease progression (66), as did pharmacological treatment with a cathepsin K inhibitor (SB-553484) in a canine model (67). Pre-clinical findings have been translated to clinical trials with the selective cathepsin K inhibitor MIV-711 reducing bone and cartilage disease progression in individuals with symptomatic, radiographic knee osteoarthritis (68).

*Chadl*, which encodes for chondroadherin-like protein, plays a role in collagen binding and in the negative regulation of chondrocyte (69). In our studies, its expression underwent the highest fold downregulation with 50 MPa hydrostatic pressure. This is consistent with a previous RNAseq study which examined the subchondral bone of patients who underwent total joint replacement due to osteoarthritis (70). In this study both *Chadl* and *Il11*, also identified in our studies, were identified as the most consistently differentially expressed genes and thus have the potential to be targeted for clinical therapies.

Whilst several ion channels known to be involved in chondrocyte mechanotransduction (e.g., *Piezo1*, *Trpv4*, *Trpv5*) (9) were unchanged in our datasets, upregulation of *Piezo2* and downregulation of *Trpm4* was observed in hip caps exposed to both magnitudes of hydrostatic pressure (**Suppl. Data 1**). Interestingly, reliable detection of *Piezo2* transcripts in primary murine chondrocytes appears to be conflicting in the literature (71, 72). *Trpm4* has been identified in cartilage samples from osteoarthritic patients (73), however its role in cartilage mechanotransduction is unclear. Downregulation of *Trpm5* and *P2rx7* was only observed in hip caps exposed to 5 MPa compared to control (**Suppl. Data 1**). This suggests that whilst our *ex vivo* models are sensitive to some changes in ion channel expression with hydrostatic pressure, other mechanisms may exist.

Our study is unique in using two different cartilage explants, both of which offer a physiological model system. We have also applied hydrostatic pressure at magnitudes based on findings from our previous meta-analysis to ensure these are representative of both physiological and injurious load (33). However, we do recognize the limitation in our sample size presented herein. Therefore, the biological interpretation of our findings should be considered appropriately, with the need for a more detailed consideration of the differences observed. For example, it would be pertinent to use a temporal approach to the application of hydrostatic pressure as in this study we applied a single load for 1 hour and is akin to a single injurious event, rather than the continual degradation seen in osteoarthritis. It would also be of further interest to utilize cartilage from an osteoarthritis model (e.g., STR/ort mouse), or ultimately from human samples, to both validate our results here, and also examine the effects of hydrostatic pressure on gene expression in disease pathology. Despite these limitations, the current study was able to statistically differentiate the effects of hydrostatic pressure on chondrocytes.

In conclusion, we identified distinct differential gene expression signatures in hip cap and metatarsal cartilage explants, indicative of the divergent phenotypes of their residing chondrocytes. Our RNAseq studies examining the cartilage response to hydrostatic pressure provided evidence for injurious hydrostatic pressure to be associated with decreases in processes including cartilage development and chondrocyte differentiation. Together this informs on the potential benefits of hydrostatic pressure in cartilage tissue engineering strategies, which need to carefully consider the magnitude of application and the effects on gene expression. Further, we identified the differential expression of a number of genes that have previously been identified as osteoarthritis risk genes, including *Ctsk* and *Chadl*, further highlighting their potential as therapeutic targets. These data will therefore contribute to a better understanding of the role of hydrostatic pressure and the chondrocyte phenotype in health and osteoarthritis.

## Data availability statement

The RNA sequencing data are available from NCBI Gene Expression Omnibus (https://www.ncbi.nlm.nih.gov/geo) under accession number GSE234112.

## Ethics statement

The animal study was approved by University of Brighton institutional review board. The study was conducted in accordance with the local legislation and institutional requirements.

## Author contributions

LB: Formal analysis, Writing – original draft, Writing – review & editing, Data curation, Investigation, Methodology. AH: Data curation, Formal analysis, Investigation, Methodology, Writing – review & editing. AS: Data curation, Formal analysis, Writing – original draft, Writing – review & editing. GB: Conceptualization, Data curation, Formal analysis, Investigation, Methodology, Writing – review & editing. PB: Conceptualization, Funding acquisition, Investigation, Methodology, Resources, Writing – review & editing. KS: Conceptualization, Formal analysis, Funding acquisition, Investigation, Methodology, Project administration, Resources, Supervision, Writing – original draft, Writing – review & editing.
