# Supplementary material for: The effects of physiological and injurious hydrostatic pressure on murine ex vivo articular and growth plate cartilage explants: an RNAseq study
Source: Front Endocrinol (Lausanne). 2023 Dec 7;14:1278596. doi: 10.3389/fendo.2023.1278596 (PMC10740163; doi:10.3389/fendo.2023.1278596)
Supplement: Supplementary file 2 [file DataSheet_2.docx]

**Suppl. Figure 1: Schematic of hydrostatic pressure application.** Samples were placed into a 5 ml capped plastic syringe (f), air bubbles removed, and placed into the chamber (e) of the steel pressure vessel (d). Following sealing the chamber with a screwed brass cap, the manual pump (a) was used to increase pressure. Following an initial period of degassing (valve c) pressure was raised within 30 secs to the required level as read from the spring dial gauge (b) and held for the required time with no pressure loss in the system. Pressure was released via the pressure release valve (c) and samples removed for subsequent incubation and analysis.


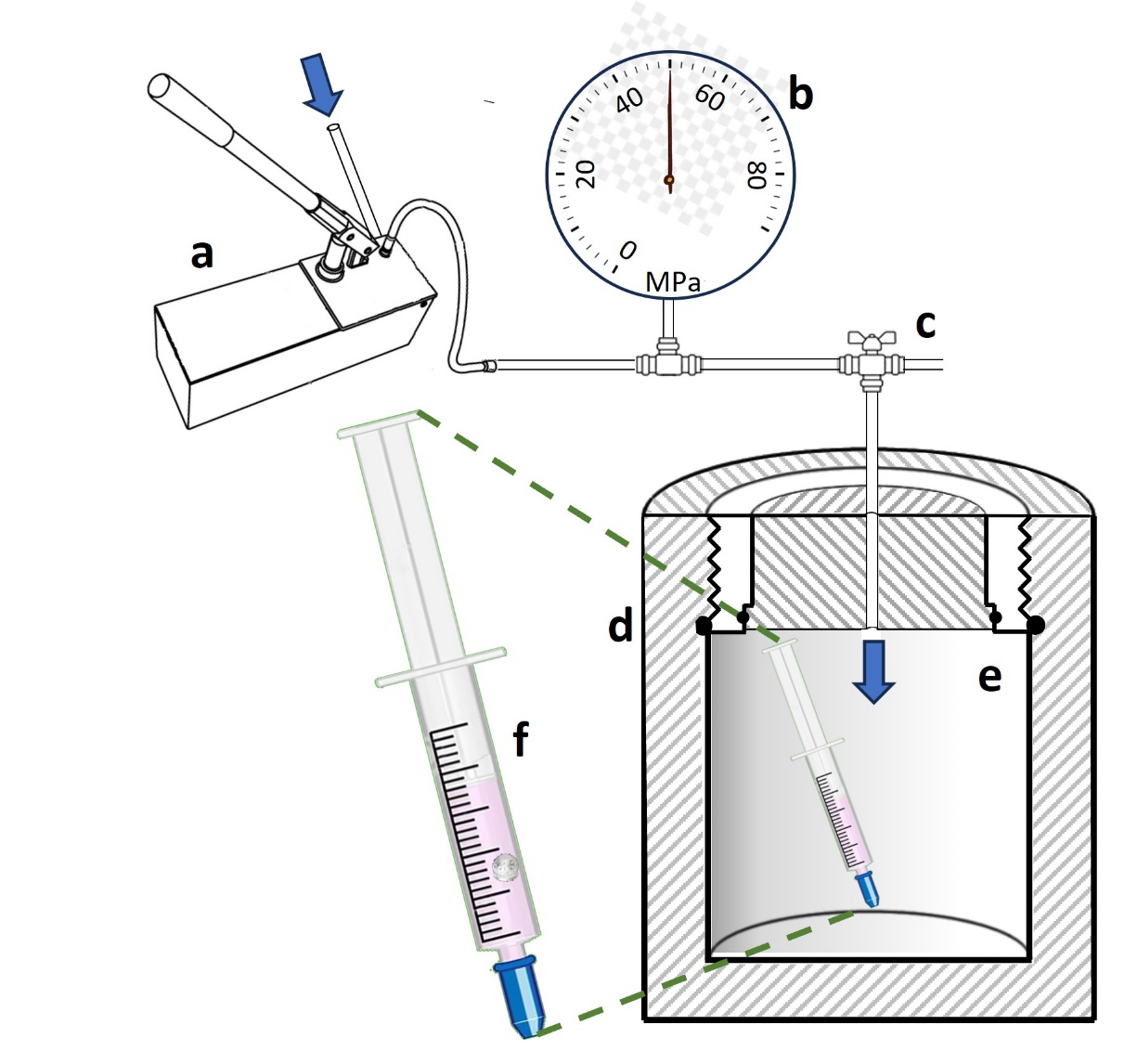


**Suppl. Figure 2: Unsupervised clustering of metatarsal samples based on their DESeq2 normalised gene-level counts**. **(A)** Heat map of inter-sample Euclidean distances, where darker blue colours indicate closer similarity. **(B)** Principal components analysis. Samples are labelled as M (metatarsal) followed by C (control – 0 MPa), 5 (5 MPa) or 50 (50 MPa) and replicate number (1-4).


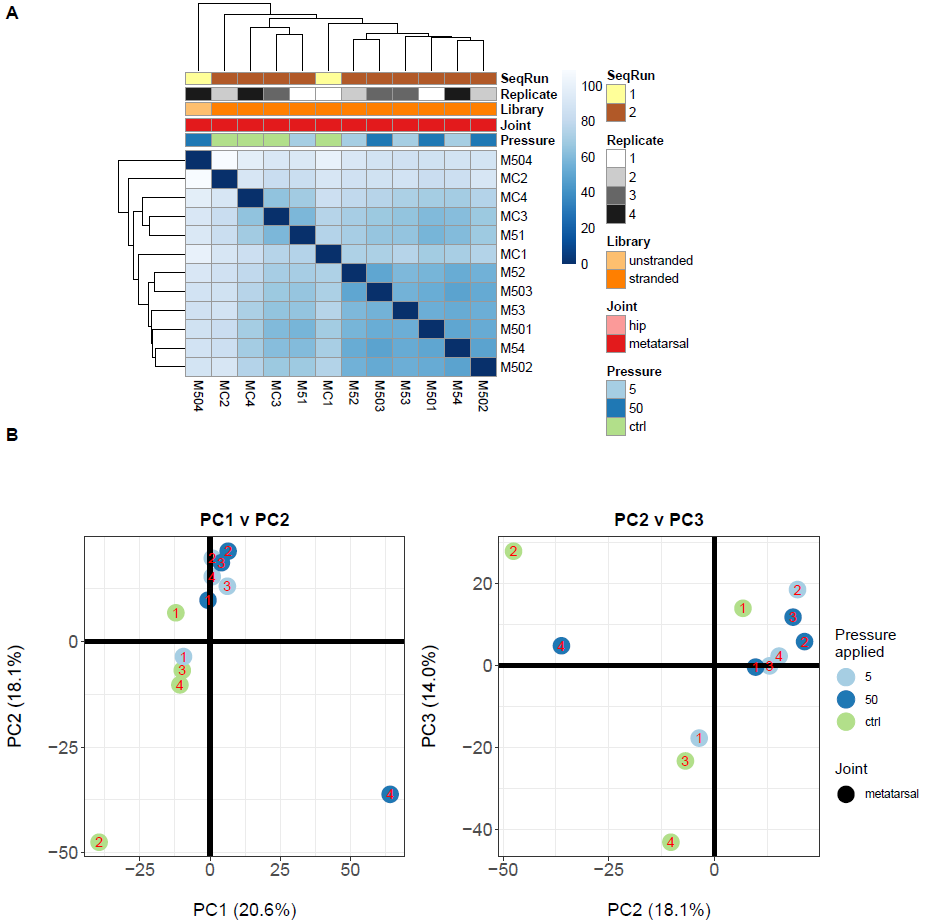


**Suppl. Figure 3: Unsupervised clustering of hip cap samples based on their DESeq2 normalised gene-level counts**. **(A)** Heat map of inter-sample Euclidean distances, where darker blue colours indicate closer similarity. **(B)** Principal components analysis. Samples are labelled as H (hip capl) followed by C (control – 0 MPa), 5 (5 MPa) or 50 (50 MPa) and replicate number (1-4).


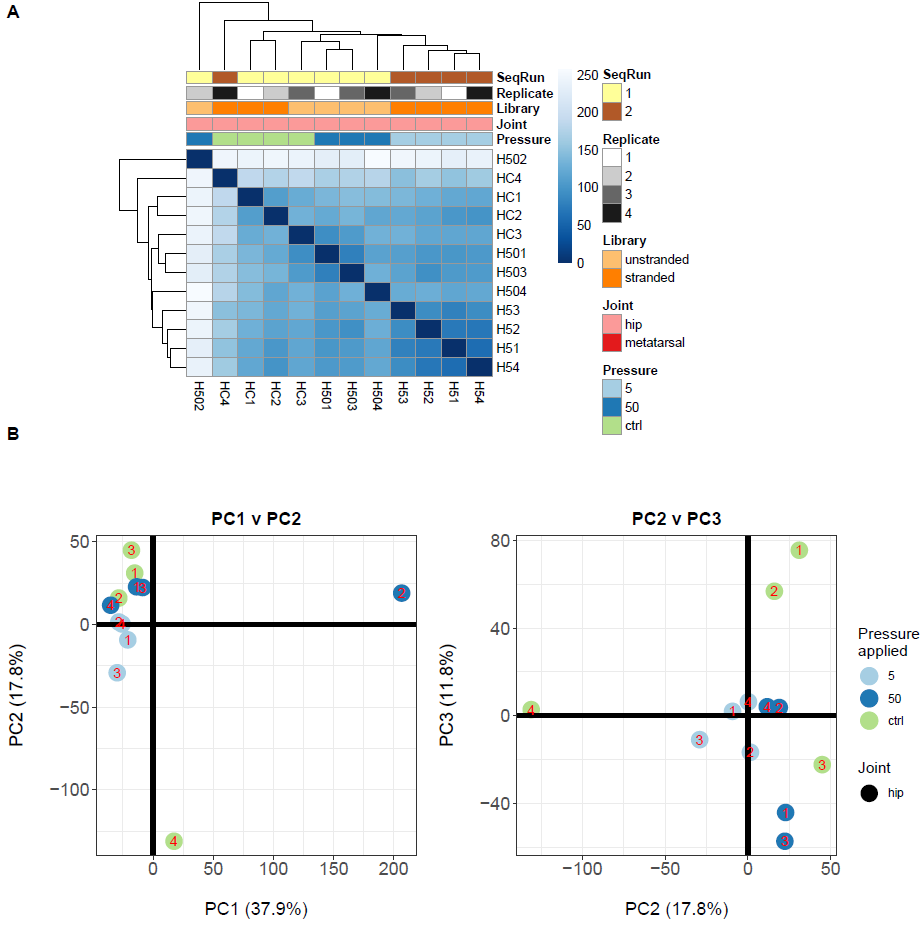


**Suppl. Figure 4:** Heat map of z-scores for genes which were differentially regulated with hydrostatic pressure in hip cap cultures and had been previously identified in recent genome-wide association studies of osteoarthritis.


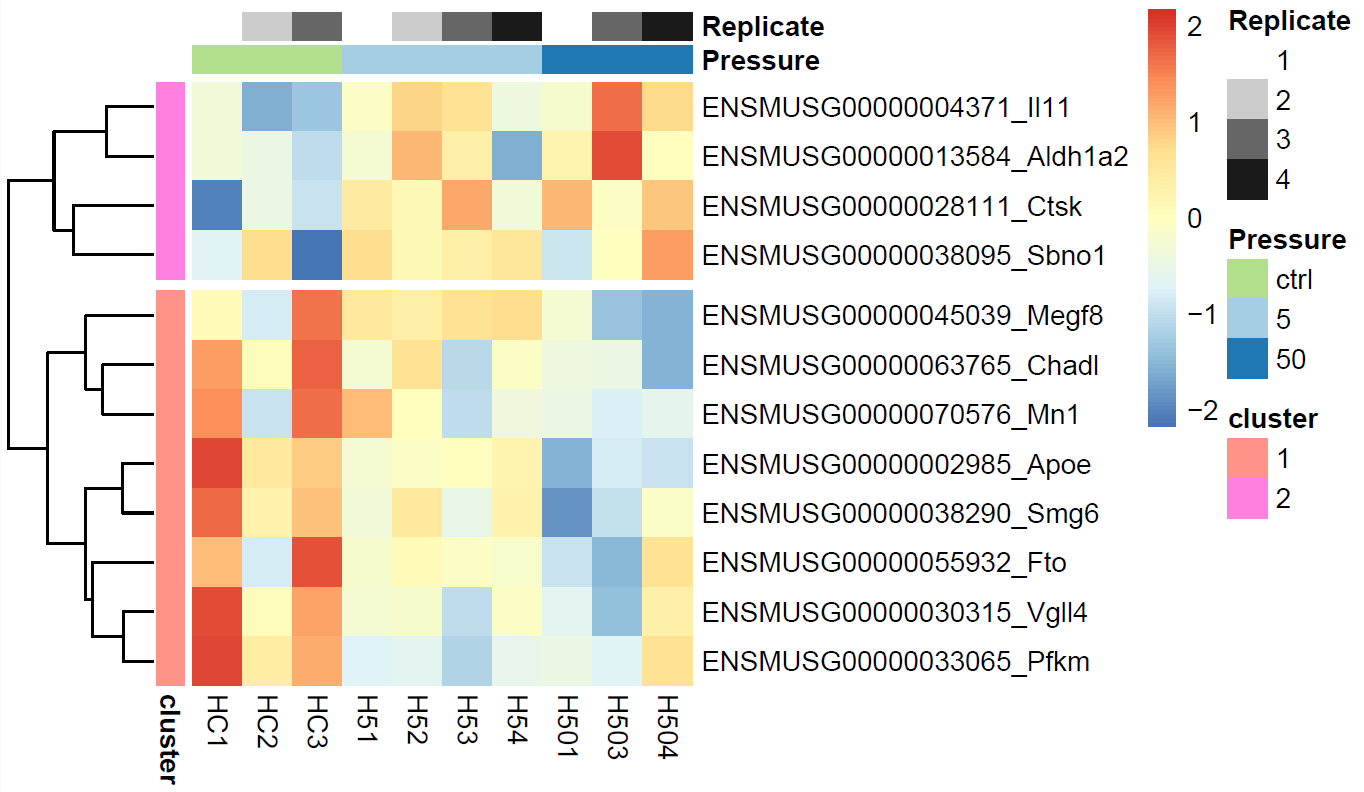


**Suppl. Table 1:** Annotations for the Gene Ontology (GO) Biological Process (BP) for hip cap versus metatarsal RNAseq datasets.

|  | **ID** | **Description** | **No. genes** | **Adjusted *p* value** |
| --- | --- | --- | --- | --- |
| Top 10 upregulated GOBP | GO:0001503 | ossification | 124 | 4.42E-36 |
|  | GO:0061448 | connective tissue development | 98 | 5.27E-33 |
|  | GO:0009100 | glycoprotein metabolic process | 96 | 1.64E-25 |
|  | GO:0030198 | extracellular matrix organization | 84 | 2.22E-21 |
|  | GO:0043062 | extracellular structure organization | 84 | 2.76E-21 |
|  | GO:0051216 | cartilage development | 83 | 3.41E-34 |
|  | GO:0060348 | bone development | 81 | 2.78E-26 |
|  | GO:0034976 | response to endoplasmic reticulum stress | 79 | 4.03E-25 |
|  | GO:0110148 | biomineralization | 62 | 8.70E-22 |
|  | GO:0002062 | chondrocyte differentiation | 55 | 1.04E-27 |
| Top 10 downregulated GOBP | GO:0050808 | synapse organization | 142 | 7.12E-34 |
|  | GO:0060537 | muscle tissue development | 140 | 6.80E-33 |
|  | GO:0042692 | muscle cell differentiation | 127 | 7.56E-30 |
|  | GO:0007409 | axonogenesis | 126 | 3.68E-25 |
|  | GO:0003012 | muscle system process | 117 | 8.83E-26 |
|  | GO:0034329 | cell junction assembly | 114 | 5.70E-23 |
|  | GO:0001655 | urogenital system development | 105 | 5.92E-23 |
|  | GO:0051146 | striated muscle cell differentiation | 97 | 5.06E-24 |
|  | GO:0050803 | regulation of synapse structure or activity | 86 | 3.29E-24 |
|  | GO:0050807 | regulation of synapse organization | 84 | 6.96E-24 |

**Suppl. Table 2:** Top 3 genes with highest upregulation and top 10 genes with highest downregulation, commonly expressed in 5 MPa vs control and 50 MPa versus control metatarsal RNAseq datasets.

|  | **5 MPa vs Control** | | | **50 MPa vs Control** | | |
| --- | --- | --- | --- | --- | --- | --- |
|  | **Gene Name** | **Log_2_ Fold Change** | **Adjusted *p* value** | **Gene Name** | **Log_2_ Fold Change** | **Adjusted *p* value** |
| Top 3 genes with highest upregulation | *Rpl9-ps4* | 29.07961 | 4.16E-11 | *Rpl9-ps4* | 28.81057567 | 3.16E-10 |
|  | *U6* | 28.21535 | 4.16E-11 | *Rps6-ps4* | 28.81057567 | 3.16E-10 |
|  | *Septin2* | 0.578819 | 8.89E-05 | *U6* | 23.33735693 | 5.77E-07 |
| Top 10 genes with highest downregulation | *Gm12671* | -27.3679 | 4.45E-05 | *Gm28661* | -28.7223197 | 9.22E-09 |
|  | *Gm10359* | -27.3679 | 4.45E-05 | *Gm28437* | -28.62386963 | 1.76E-12 |
|  | *Rpl10a-ps1* | -26.88 | 7.02E-05 | *Gm28439* | -28.22142394 | 3.49E-05 |
|  | *Gm10052* | -26.5386 | 8.89E-05 | *Gm12671* | -27.4399153 | 6.68E-05 |
|  | *Rps6-ps4* | -26.2024 | 1.90E-08 | *Gm10359* | -27.4399153 | 6.68E-05 |
|  | *Rpl21-ps15* | -25.1948 | 0.00028 | *Rpl21-ps15* | -26.73154565 | 0.00013 |
|  | *Gm10222* | -25.0437 | 0.0003 | *Rpl10a-ps1* | -25.67384565 | 0.00031 |
|  | *Rps12-ps3* | -24.1107 | 0.00013 | *Rps12-ps3* | -23.1337769 | 0.00045 |
|  | *Endou* | -2.16479 | 0.01453 | *Gm10052* | -22.64342445 | 0.00173 |
|  | *Gsg1l* | -0.56493 | 0.00055 | *Gm10222* | -19.88815427 | 0.00427 |

**Suppl. Table 3:** Top 2 genes with highest upregulation and greatest downregulation that are uniquely expressed in 5 MPa versus control and 50 MPa versus control in the metatarsal RNAseq dataset.

|  | **5 MPa vs Control** | | | **50 MPa vs Control** | | |
| --- | --- | --- | --- | --- | --- | --- |
|  | **Gene Name** | **Log_2_ Fold Change** | **Adjusted *p* value** | **Gene Name** | **Log_2_ Fold Change** | **Adjusted *p* value** |
| Top 2 genes with highest upregulation | *Gm15516* | 10.43418 | 0.024787 | *Gm6344* | 33.29918 | 1.97E-07 |
|  | *Zfp990* | 2.466429 | 0.001984 | *Bloc1s5* | 0.299111 | 0.034986 |
| Top 2 genes with highest downregulation | *Gm5537* | -29.2388 | 2.54E-15 | *Rpl36a-ps2* | -4.57593 | 0.018639 |
|  | *Gm10443* | -21.9814 | 4.16E-11 | *Gm32017* | -0.3286 | 0.034986 |

**Suppl. Table 4**: Top 10 genes with highest upregulation and top 10 genes with highest downregulation, commonly expressed in 5 MPa versus control and 50 MPa versus control in the hip cap RNAseq datasets.

|  | **5 MPa vs Control** | | | **50 MPa vs Control** | | |
| --- | --- | --- | --- | --- | --- | --- |
|  | **Gene Name** | **Log_2_ Fold Change** | **Adjusted *p* value** | **Gene Name** | **Log_2_ Fold Change** | **Adjusted *p* value** |
| Top 10 genes with highest upregulation | *mt-Co2* | 3.861770644 | 1.20E-10 | *Gm49394* | 13.50342072 | 2.41E-05 |
|  | *mt-Atp6* | 3.511212933 | 0.000187849 | *Gm12191* | 9.194177325 | 3.32E-05 |
|  | *mt-Co3* | 3.457740125 | 0.000145902 | *Gm45062* | 6.85408364 | 0.000910969 |
|  | *mt-Nd3* | 3.224000243 | 0.000100063 | *Gemin4* | 5.045475205 | 7.18E-05 |
|  | *mt-Nd1* | 3.096226408 | 0.000376173 | *mt-Nd3* | 4.026272198 | 1.39E-07 |
|  | *mt-Co1* | 2.74274834 | 0.000400982 | *mt-Nd2* | 3.745065618 | 0.000106936 |
|  | *Nutf2-ps1* | 2.649443999 | 0.049036319 | *mt-Nd1* | 3.620577046 | 1.24E-05 |
|  | *mt-Nd5* | 2.51981988 | 0.00061336 | *mt-Co1* | 3.360772144 | 7.32E-06 |
|  | *mt-Nd4* | 2.484054695 | 0.001486832 | *mt-Atp6* | 3.354536124 | 8.34E-05 |
|  | *Ifitm3* | 2.467004261 | 0.000214404 | *mt-Co2* | 3.299205031 | 3.92E-09 |
| Top 10 genes with highest downregulation | *Snhg4* | -3.486073226 | 0.011386169 | *Snhg4* | -3.83043866 | 0.006048404 |
|  | *Hoxd8* | -2.343113938 | 0.000377062 | *Lama2* | -3.153301518 | 6.49E-10 |
|  | *Mir540* | -2.265276889 | 0.000449718 | *Olfr57* | -2.108403829 | 0.000357334 |
|  | *Gm49394* | -2.012936641 | 0.002620513 | *Mir540* | -2.036317497 | 0.000246019 |
|  | *Scn8a* | -2.010893711 | 0.014905645 | *Gm11175* | -1.714214415 | 0.001641725 |
|  | *Ccdc59* | -1.927970123 | 6.71E-05 | *Ccdc59* | -1.612781032 | 3.65E-05 |
|  | *Lama2* | -1.65371248 | 0.00055755 | *Thnsl2* | -1.556200081 | 0.037482276 |
|  | *Gm11175* | -1.452316526 | 0.008304903 | *Tmem8b* | -1.467009907 | 0.030936955 |
|  | *Thnsl2* | -1.422750563 | 0.037729217 | *Caskin1* | -1.379721524 | 0.000300418 |
|  | *Pfdn5* | -1.303381065 | 0.034377932 | *Scn8a* | -1.319270897 | 0.000726646 |

**Suppl. Table 5:** Differential expression of osteoarthritis risk genes identified in recent genome-wide association studies in our hip cap versus metatarsal datasets.

|  | **Gene Name** | **Log_2_ Fold Change** | **Adjusted *p* value** |
| --- | --- | --- | --- |
| Upregulated genes | *Col2a1* | 4.460767991 | 1.30649E-21 |
|  | *Vdr* | 3.971054306 | 1.16306E-10 |
|  | *Ecrg4* | 3.888023858 | 1.63708E-25 |
|  | *Chadl* | 3.768896511 | 2.52913E-18 |
|  | *Fgfr3* | 3.297154052 | 1.42335E-16 |
|  | *Wscd2* | 3.211908489 | 2.58119E-18 |
|  | *Mgp* | 2.803270536 | 1.34854E-19 |
|  | *Col11a1* | 2.498613837 | 5.73403E-10 |
|  | *Maml2* | 2.468286613 | 1.32893E-16 |
|  | *Pthlh* | 2.438911388 | 4.54864E-06 |
|  | *Tgfb1* | 2.340071354 | 2.50821E-21 |
|  | *Runx2* | 2.222281426 | 9.68529E-20 |
|  | *Hfe* | 2.166828101 | 4.70158E-08 |
|  | *Rnf144b* | 2.0411437 | 1.9187E-11 |
|  | *Myo6* | 2.029292116 | 7.45078E-11 |
|  | *Col27a1* | 1.949935641 | 2.15522E-06 |
|  | *Slc39a8* | 1.805861691 | 2.89549E-07 |
|  | *Apoe* | 1.758758984 | 2.72816E-10 |
|  | *Tln2* | 1.753667878 | 3.3663E-08 |
|  | *Wwp2* | 1.606707462 | 6.09549E-07 |
|  | *Tnfsf11* | 1.478468423 | 0.00744417 |
|  | *Hdac9* | 1.463218151 | 0.00120221 |
|  | *Nog* | 1.401890629 | 0.003514769 |
|  | *Snap47* | 1.347139689 | 4.96389E-09 |
|  | *Nr3c1* | 1.238205436 | 7.50369E-10 |
|  | *Tnc* | 1.199109013 | 0.017091841 |
|  | *Wnt10b* | 1.142415309 | 0.008922547 |
|  | *Ptch1* | 1.078573351 | 7.56062E-05 |
|  | *Tgfa* | 0.981986875 | 0.009376428 |
|  | *Megf8* | 0.902555936 | 3.60765E-07 |
|  | *Tsen15* | 0.830409988 | 0.000844041 |
|  | *Slc44a2* | 0.78238992 | 4.77057E-05 |
|  | *Cradd* | 0.766375282 | 0.018328221 |
|  | *Pfkm* | 0.679972085 | 0.031032939 |
|  | *Fto* | 0.630807235 | 0.001744789 |
|  | *Smo* | 0.44611072 | 0.016041563 |
| Downregulated genes | *Fbn2* | -6.181002214 | 3.03739E-22 |
|  | *Aldh1a2* | -4.347075419 | 8.95345E-05 |
|  | *Lmx1b* | -3.511375433 | 5.20141E-19 |
|  | *Fgf18* | -3.20392901 | 6.71533E-07 |
|  | *Tbx4* | -2.968051536 | 2.2852E-37 |
|  | *Gdf5* | -2.488862978 | 2.67274E-08 |
|  | *Tsku* | -2.143277684 | 2.07688E-08 |
|  | *Chrm2* | -2.13087544 | 0.012916191 |
|  | *Astn2* | -2.035862011 | 0.000634154 |
|  | *Sh3pxd2b* | -2.027616451 | 1.9909E-10 |
|  | *Bmp5* | -1.99457731 | 0.000304998 |
|  | *Twist1* | -1.682934037 | 1.42349E-07 |
|  | *Chst3* | -1.524199572 | 3.39685E-08 |
|  | *Chst3* | -1.524199572 | 3.39685E-08 |
|  | *Triobp* | -1.055776456 | 0.000382675 |
|  | *Mapt* | -1.051104969 | 0.04581733 |
|  | *Nmral1* | -0.830244729 | 0.016896225 |
|  | *Pik3r1* | -0.733466659 | 0.005863583 |
|  | *Ltbp1* | -0.687704627 | 0.006790104 |
|  | *Sbno1* | -0.545263771 | 0.033567079 |
|  | *Mbd1* | -0.448776156 | 0.026937292 |
